# Learning the Regulatory Code of Alzheimer's Genomes

**DOI:** 10.1002/alz70855_103323

**Published:** 2025-12-24

**Authors:** Towfique Raj

**Affiliations:** ^1^ Icahn School of Medicine at Mount Sinai, New York, NY, USA

## Abstract

**Background:**

Investigating the role of rare genetic variants in Alzheimer's disease (AD) is challenging due to the limited specificity of functional predictions in brain cell types. To address this, we developed brain cell‐type‐specific non‐coding variant prediction scores, incorporating splicing and enhancer/promoter effects. We also introduced a novel rare variant testing method and applied these tools to whole‐genome sequencing (WGS) data from the Alzheimer's Disease Sequencing Project (ADSP).

**Method:**

We trained deep learning models on microglia RNA‐seq data to predict variant effects on splicing and gene regulation. Additionally, we developed a splicing modifier score (SMS) to refine the prioritization of fine‐mapped splicing QTL variants. To improve rare variant discovery, we implemented a Bayesian probabilistic model that assigns global, trait‐specific weights to functional annotations. We applied this to WGS data from 7,966 AD cases and 13,412 controls to identify novel AD‐associated genes and regulatory annotations.

**Result:**

Our models outperformed baseline approaches in predicting microglia splicing, achieving a PR‐AUC of 0.853. The delta scores accurately predicted fine‐mapped sQTL variants with a ROC‐AUC of 0.656, and MPRA validation in iPSC‐derived microglia confirmed functional effects for several AD‐associated variants. Using our Bayesian model, we identified 10 novel genetic associations undetected by standard rare variant methods, with splicing annotations showing the strongest enrichment for AD‐associated non‐coding rare variants.

**Conclusion:**

We introduce a comprehensive framework integrating functional annotations, splicing and enhancer/promoter variants, and brain cell‐type‐specific rare variant analysis. This approach enhances genome‐wide rare variant association tests, improving the identification of AD‐relevant genes and regulatory elements with greater specificity. Furthermore, our method uncovers previously unidentified rare variant associations in AD, providing promising targets for future functional validation and therapeutic development.

